# Breast-torso movement coordination during running in different breast support

**DOI:** 10.1038/s41598-024-71337-y

**Published:** 2024-09-12

**Authors:** Genevieve K. R. Williams, Jo Reeves, Domenico Vicinanza, Chris Mills, Brogan Jones, Joanna Wakefield-Scurr

**Affiliations:** 1 Faculty of Health and Life Sciences, University of Exeter, Exeter, UK; 2 Faculty of Science and Engineering, Anglia Ruskin University, Cambridge, UK; 3 School of Sport, Health and Exercise Science, University of Portsmouth, Portsmouth, UK

**Keywords:** Breast, Biomechanics, Coupling, Nonlinear dynamics, Female, Bra, Physiology, Anatomy

## Abstract

To reduce breast motion with a bra, we need to understand what drives the motion of the breasts, and what variables change as support increases. Quantifying breast-torso coordination and movement complexity across the gait cycle may offer deeper insights than previously reported discrete time lag. We aimed to compare breast-torso coordination and mutual influence across breast support conditions during running. Twelve female participants ran on a treadmill at 10 km h^−1^ with an encapsulation and compression sports bra, and in no bra. Nipple and torso position was recorded. Vector coding, granger causality and transfer entropy were calculated within gait cycles. In both bra conditions, a greater percentage of gait cycles was spent with the breast and torso in-phase (> 90%) compared to no bra running (~ 66%, p < 0.001), with most time spent in-phase in the encapsulation versus compression bra (p = 0.006). There was a main effect of breast support condition on Granger causality (p < 0.001), both from breast to torso and torso to breast. Transfer of information was highest from torso to breast, compared to breast to torso in all conditions. Overall, these results provide novel insight into the mutual and complex interaction between the breast and the torso while running in different bra conditions. The approaches presented allow for a greater understanding of bra support conditions than existing discrete measures, which may relate to comfort and performance. Therefore, measures of coupling, predictability and transfer of complexity should be employed in future work examining these features.

## Introduction

Coupling between two oscillators captures their relative motion in the presence of external drivers and external constraints. Previous research shows that sports bras are effective in altering the kinematics of the breast^1^ and the body^2,3^ when studied in isolation. However, the dynamic interaction between the breast and the torso during running has not been assessed. In order to further understand the fundamentals of how and when a sports bra affects breast motion, this paper quantifies coupling between the breast and torso when running in different breast support conditions. Coupling was quantified based on relative motion of the breast, mutual predictability of motion and transfer of complexity.

During running breast-torso coordination has been quantified as a time-lag between the breast and torso position at discrete time points of the gait cycle^4,5^. The time-lag, defined at maximum superior displacement during the flight phase of running, was identified as the objective variable with the greatest difference (effect size) between breast support conditions^4^. A time-lag between the breast and the torso at maximum inferior displacement after foot contact during bare-breasted no-bra running may result in what has been described as a “slap” of the breast onto the chest, which may be related to breast pain in running^6^.

Time-lag at discrete points in the gait cycle provides some indication of the coupling or coordination between the breast and torso, however other potentially relevant instances related to gait cycle events are yet to be considered. Moreover, it could be falsely concluded that no differences exist between conditions (in this case bra support), if differences are examined only at discrete time points and not over the entire gait cycle. To overcome these limitations, methods to formally calculate the relative phase between the continuous motion of oscillators are well-established^7^. Vector coding has the advantage of being a simple technique that provides inherently interpretable information to quantify the coordination between pairs of oscillators over an entire time series^7–12^, and has been applied in a number of different scenarios, particularly in gait analysis^8,9,12–17^. Overall, vector coding angles can be divided into four coordination patterns, or frequency bins of 45° (Fig. 4)^8^. The four frequency bins represent coordination patterns: in-phase; anti-phase; proximal phase dominancy (proximal segment leading) and distal phase dominancy (distal segment leading)^8,9,15,18^. Based on evidence from work by Risius et al.^4^, we would expect that reduced time lag with increased bra support conditions implies more in-phase coupling between the breast and torso. Examining the coordination between the breast and torso through the entire running gait cycle using vector coding will offer insights into the fundamentals of breast movement and the support requirements and/or performance of sports bras, particularly as the timing of peak breast skin strain in each region of the breast varies across the gait cycle^1^.

Coordination in movement patterns provides one view on coupling between the breast and torso. Since the breast and the torso are mechanically linked, they have an effect on each other. In understanding coupling through this mutual influence we took two approaches. Firstly, Granger causality^19^ is a technique that measures the mutual influence, testing how the knowledge of one signal can help improve the prediction of another, specifically, if the history of signal X contains information to predict signal Y beyond the information contained in the history of signal Y alone, then there is a predictive relationship. External support of a sports bra constrains the movement of the breast relative to the torso. For example, compression bras limit breast movement by compressing and flattening the breasts onto the torso^20^, whereas encapsulation sports bras have moulded cups that support each breast separately^20,21^. Compression and encapsulation bras have demonstrated 51% and 59% reductions in breast displacement in running, respectively, relative to no bra^22^. On the other hand, no significant difference in torso kinematics was reported with increased breast support during running^4^, while torso movement variability has been shown to increase^3^. Therefore, based on previous work, it is likely that prediction of the breast motion during dynamic activity will be improved with knowledge of the torso motion in more supportive bra conditions compared to no bra or less supportive conditions. Quantifying this effect could inform modelling studies that have previously modelled the torso as the suprasternal notch providing the driving force to the breast^23^, as well as informing performance aspects of bra design.

Secondly, complexity of movement has been related to health status^24^ and may also be an important factor in comfort in breast motion as breast movement has been described as more “erratic” In water, in which movement was more comfortable, compared to land^25^. Transfer entropy is based on Shannon’s information theory and the complexity of information contained in the signal, and is used to quantify to what degree complexity contained in the torso signal is transferred to the breast signal and vice versa. Thus, the complexity of the torso motion that is transferred to the breast may be an aspect of coupling related to comfort. Quantifying the transfer entropy between the torso and breast provides information that transcends the current mechanical understanding of breast motion, by establishing the extent to which signal complexity is transferred between the two systems.

The aim of this study was to quantify and compare breast-torso coordination and mutual influence as a function of sports bra type during running. Although breast displacement in running is multi-planar, for simplicity, only vertical displacement was analysed. Nonetheless, previous work has shown that at speeds of 10 km/h and above the vertical component accounts for around 50% of total displacement^22^. Based on previous literature which shows that vertical breast displacement reduces from no bra running to running in a compression sports bra and finally to running in an encapsulation sports bra; it was hypothesised that (1) based on vector coding, the mean phase angle between the breast and torso would be more in-phase with increased breast support across the running cycle; (2) based on Granger causality, in more supportive bra conditions, knowledge of torso motion will improve prediction of breast motion more than in less supportive bra conditions, and (3) based on transfer entropy, the level of breast support will affect the transference of signal characteristics from one system (torso or breast) to the other system, and that this transference will increase as breast support increases.

## Results

### Time lag

**Fig. 1:**
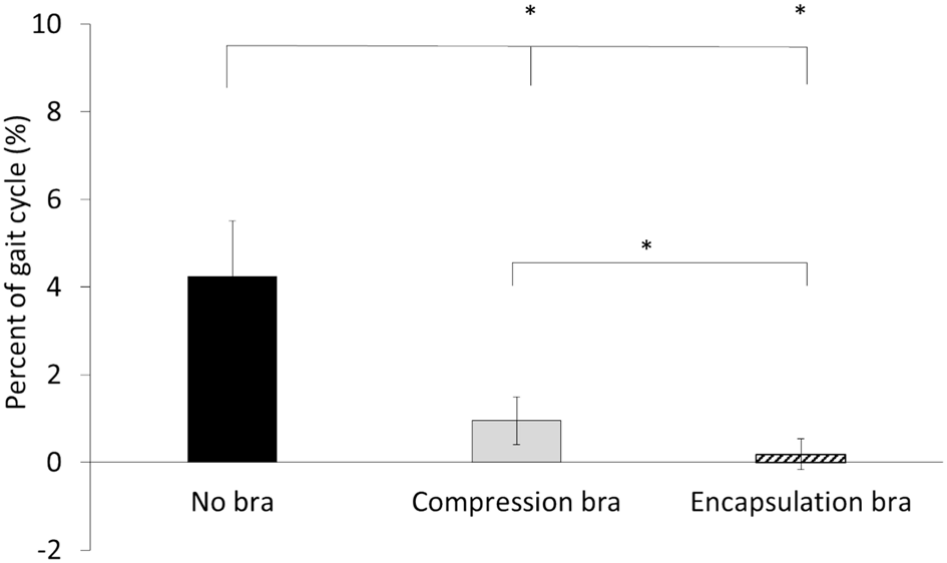
Mean (SD) time lag between maximum nipple position and maximum sternum position as a percentage of the gait cycle of the left nipple in no bra, compression bra and encapsulation bra.

There was a main effect of bra support condition on discrete time lag (Fig. 1, (p < 0.001). Post hoc comparisons revealed a significantly greater time lag in the no bra compared to both bras (p < 0.001) and a greater time lag in the compression bra than the encapsulation bra (p < 0.001).

### Vector coding

**Fig. 2:**
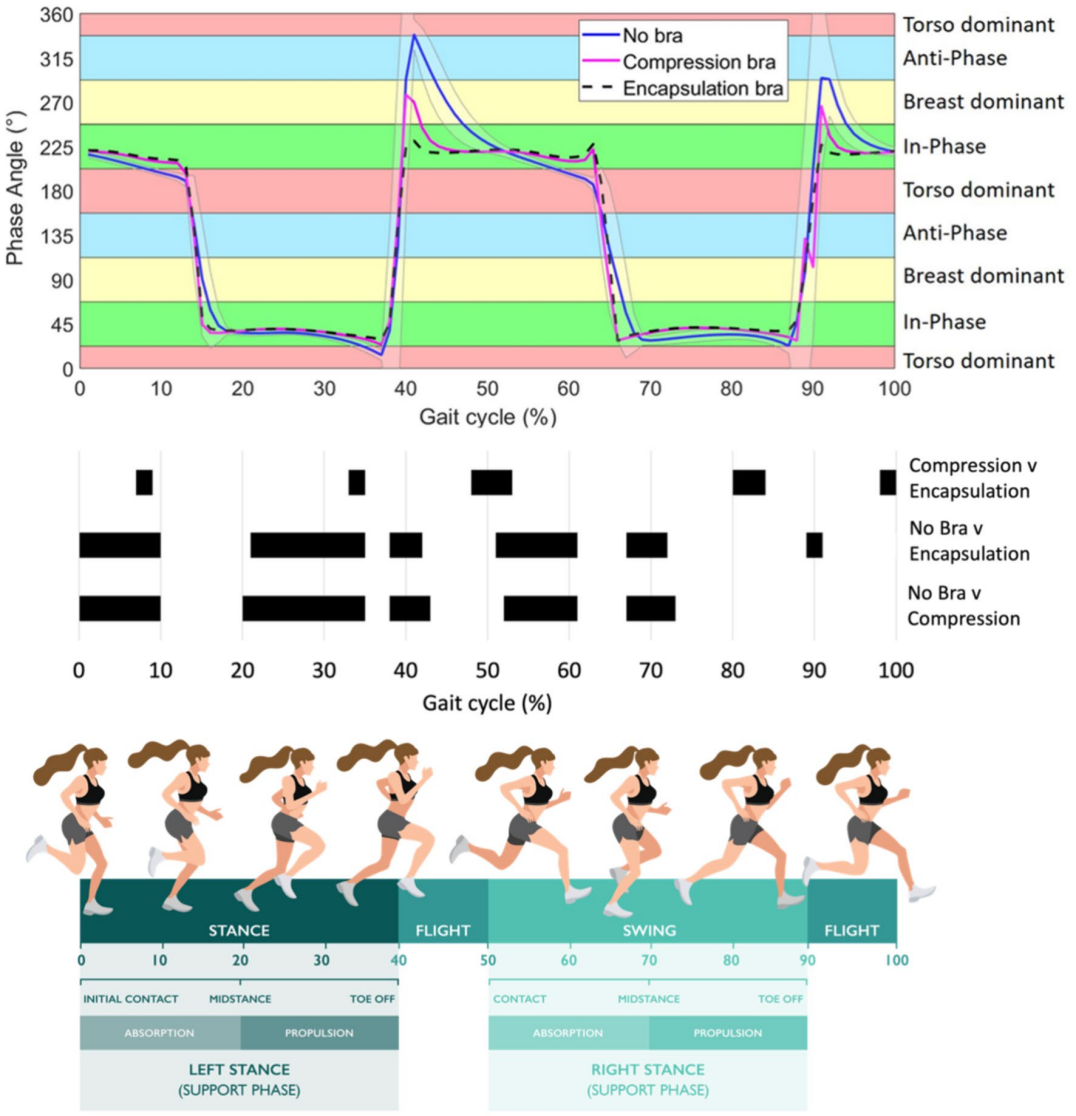
Top: Mean vector coding (phase angle) across the gait cycle in each condition for 12 participants. Shaded area around the blue line represents one standard deviation above and below the mean of the no bra condition. Middle: Post-hoc results from SPM analysis, where shaded areas represent significant difference in phase angle between conditions. Bottom: Illustration of corresponding phases of the gait cycle and terminology used to describe each phase.

In line with Eq. (1), Figs. 4 and 6, Vector Coding was performed to quantify the phase relationship between the breast and torso motion in the vertical direction throughout the entire gait cycle. In Fig. 2 it can be seen that during absorption with no bra the breast and the torso move from in-phase to torso dominancy, while with both bras the breast and the torso remain in-phase. The SPM ANOVA analysis found a significant main effect of bra support on mean phase angle across much of the gait cycle. During the first part of the stance phase on each foot (absorption) there was a significant difference in mean phase angle between the no bra and both of the bra conditions. Although there was a significant difference between no bra and both the bra conditions during the propulsion period of stance on each foot, the breast and torso remained in-phase during this period (Fig. 2). There was also a notable difference in mean phase angle between no bra and both sports bras at ~ 40% and ~ 90% of the gait cycle (corresponding to takeoff with the left and right foot respectively, Fig. 2). The difference between conditions was however greater at ~ 40% compared to ~ 90% (Fig. 2). At takeoff in the no bra condition there was a transition from in-phase coupling to anti-phase coupling, indicating that the breast and torso were moving in opposite directions, i.e. when the torso reached the apex of flight and then began descending, the breast was still ascending. However, the breast and the torso remained in-phase during the entire flight phase with the encapsulation bra. While the differences in phase angle between bras was smaller than between each bra and no bra, these differences occurred at similar points of the gait cycle (Fig. 2).

**Fig. 3:**
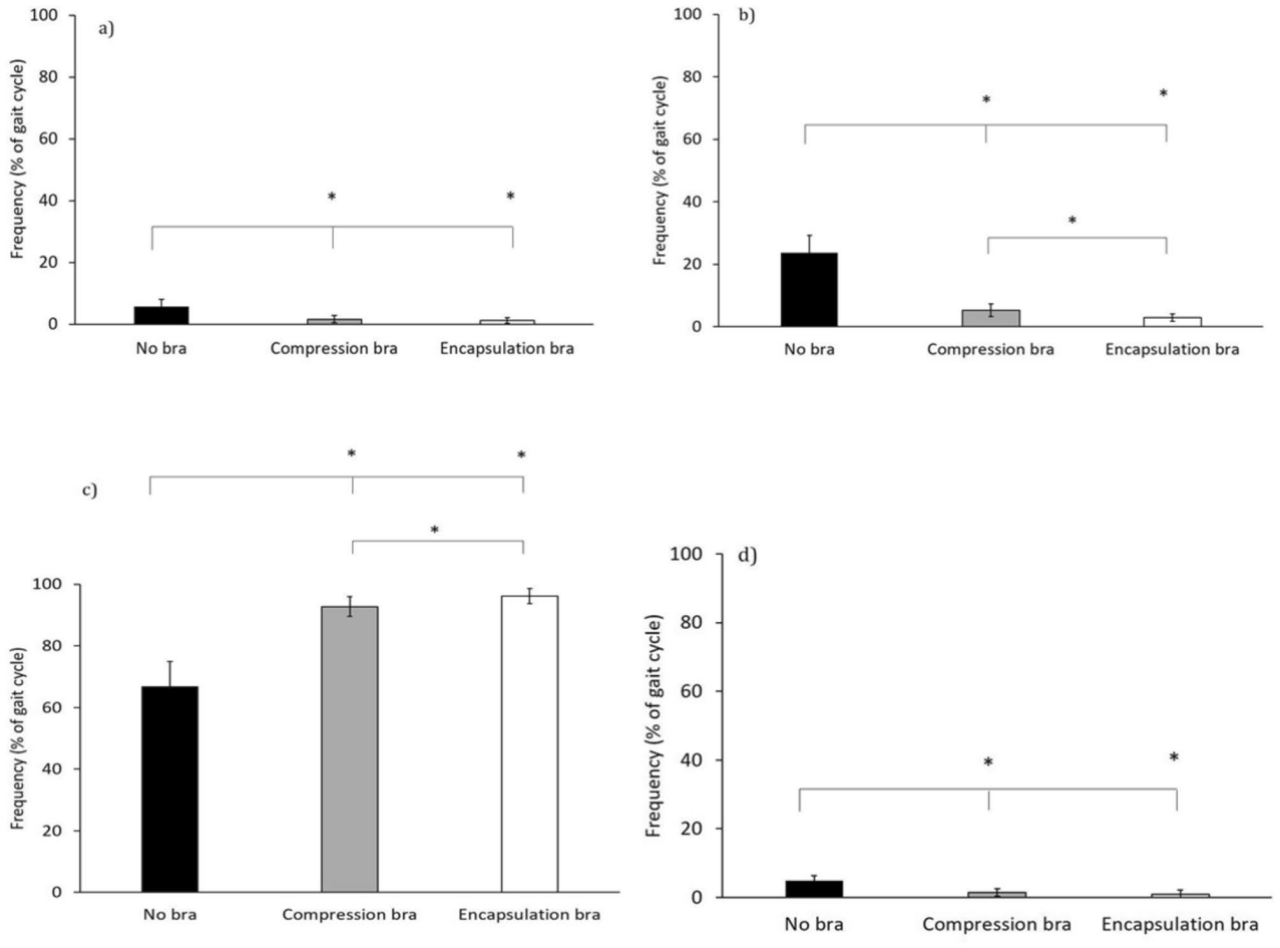
Coordination pattern frequency for breast-torso coordination (**a**) Breast dominant; (**b**) Torso dominant; (**c**) In-phase and (**d**) Anti-phase. Black bars represent the no bra condition, grey bars represent the compression sports bra condition and white bars represent the encapsulation sports bra condition, *significant difference (p < 0.05).

The proportion of the gait cycle in which the breast and torso were in each coordination pattern were quantified. There was a main effect of bra support on the percentage of time spent in each coordination bin (p < 0.001). Post hoc comparisons revealed significantly less breast dominancy (Fig. 3a, p < 0.001) and anti-phase coordination (Fig. 3d, p < 0.001) in both bra conditions compared ore in-phase coordination in both bras (> 90% of the gait cycle) compared to no bra (67% of the gait cycle, Fig. 3c, p < 0.001) and more in-phase coordination in the encapsulation bra (96% of the gait cycle) compared to the compression bra (93% of the gait cycle, p = 0.006). There was less torso dominancy in both sports bras compared to no bra (Fig. 3b, p < 0.001) and there was significantly more torso dominancy in the compression bra than the encapsulation bra (p = 0.001).

### Granger causality

There was a main effect of breast support condition on Granger causality (p < 0.001) both from breast to torso and torso to breast (Table 1). Post hoc tests revealed significant differences between all conditions [Breast to torso: No bra compared to compression (p < 0.001) and encapsulation (p < 0.001) bra, and encapsulation to compression bra (p = 0.001). Torso to breast: No bra compared to compression (p < 0.001) and encapsulation (p = p < 0.001) bra, and encapsulation to compression bra (p < 0.001)].

**Table 1:** Mean and standard deviation of Granger causality values in direction breast to torso and torso to breast for the three bra support conditions (no bra, compression bra and encapsulation bra).

Granger causality					
Breast → Torso			Torso → Breast		
No bra	Compression bra	Encapsulation bra	No bra	Compression bra	Encapsulation Bra
Mean (SD)	Mean (SD)	Mean (SD)	Mean (SD)	Mean (SD)	Mean (SD)
1.24 (0.36)	0.41 (0.21)	0.09 (0.5)	1.01 (0.28)	0.40 (0.20)	0.09 (0.05)

### Transfer entropy

Transfer of information was highest from the torso to the breast, compared to the breast to the torso in all breast support conditions (Table 2). Transfer of information from the breast to the torso, a significant main effect of breast support condition (p = 0.041) was observed. Post hoc tests revealed significant differences between the no bra conditions and the compression (p = 0.040) and encapsulation (p = 0.044) bra conditions. No main effect of bra condition was observed in the direction of torso to breast (p = 0.876).

**Table 2:** Mean and standard deviation transfer entropy values in direction breast to torso and torso to breast for the three bra support conditions (no bra, compression bra and encapsulation bra).

Transfer entropy					
Breast → Torso			Torso → Breast		
No bra	Compression bra	Encapsulation bra	No bra	Compression bra	Encapsulation Bra
Mean (SD)	Mean (SD)	Mean (SD)	Mean (SD)	Mean (SD)	Mean (SD)
0.005 (0.004)	0.011 (0.005)	0.013 (0.009)	0.036 (0.010)	0.034 (0.018)	0.043 (0.020)

## Discussion

The aim of this study was to quantify and compare breast-torso coordination and mutual influence as a function of sports bra support in running. In line with our first hypothesis, mean phase angle between the breast and torso was more in-phase across the running cycle with increased breast support. Contrary to hypothesis 2 while knowledge of breast motion improved predictability of torso motion and vice versa with no bra and with the compression bra, this was not the case in the most supportive condition (encapsulation bra). Thirdly, in contrast to hypothesis 3, complexity transferred from the breast and torso was significantly higher in the bra compared to no bra conditions but not significantly affected by breast support condition. This transfer of complexity between breast and torso implies that in breast support conditions, some of the dynamical characteristics of one affect the inner dynamics of the other.

There was an effect of sports bra support condition on phase angle across the gait cycle, advocating the value of examining continuous breast-torso coordination offered by vector coding techniques. While the breast and torso were in-phase for approximately two thirds of the gait cycle when running in no bra, in both sports bras the breast and torso were in-phase for over 90% of the gait cycle and almost entirely in-phase with the encapsulation sports bra. The effect of bra condition was particularly evident at 40% and 90% of the gait cycle, at approximately take-off with the left foot and right foot respectively, after which there is a transition to the flight phase, where both legs are off the ground. In both sports bra conditions there was a transition from in-phase throughout stance to another state of in-phase coordination in the flight phase. By contrast during no bra running, the breast and torso were barely in-phase in the entire flight phase of the gait cycle, and only in-phase during the second half of stance (the propulsion period^26^). The greater proportion of in-phase coordination between the breast and torso throughout the flight phase when running with breast support than without, extends the finding of previous work which showed an effect of breast support on the discrete time-lag between the breast and the torso reaching maximum vertical displacement in the flight phase of running^4^. The greater difference in coordination of the left nipple and torso seen at 40% of the cycle (left step) compared to 90% of the cycle (right step), approximate left and right foot take-off respectively, emphasises the importance of identifying the contralateral foot contact when evaluating breast motion.

Regarding the stance phase, during the first half of stance—the absorption period^26^—there was a large difference in phase angle between either bra and the no bra. During this time there was a period of torso dominance when running in no bra, corresponding to when the breasts abruptly decelerate after the torso, causing the breast to “slap” down on the torso^6^. While discrete measures may suggest differences between bra support conditions at a single time point, this investigation demonstrates that vector coding can quantify differences in breast-torso behaviour with respect to functionally relevant phases of the gait cycle, such as foot contact and toe-off.

By using vector coding to calculate the phase angle between the breast and the torso we can assess the effect of different bra support in the context of the gait cycle, which could inform bra design. Our results demonstrated that the high support encapsulation bra produced more in-phase coordination between the breast and torso compared to the compression bra, while more torso dominance was observed in the compression bra compared to the encapsulation bra. The finding that the breast and torso were almost entirely in-phase across the gait cycle with the encapsulation bra, provides evidence of a ‘locking’ mechanism to move the breast in line with the torso. Indeed, a fundamental function of sports bras is to reduce the inertia components of the breast motion^4^. Since the mid-flight phase and take off phase distinguished the two bra conditions it is suggested that future investigations into sports bra performance or comfort consider the relative phase of breast and torso motion throughout the gait cycle to better define the effects of different forms of breast support and better explain changes in predefined sports bra performance or comfort ratings.

To determine the mutual influence between the torso and the breast during running, we provide quantitative evidence of the degree to which motion variability in the breast can be better predicted with knowledge of the torso, and vice-versa, in the different breast support conditions. This is a powerful technique to use in breast biomechanics research, adding to the quantification of phase relationships, because it shows the increase in predictability of breast motion (reduction of uncertainty) when the motion of the torso is taken into account. For example, in the case of the encapsulation bra, because of the tight coupling between the breast and bra there is little new information from the torso motion signal to predict breast motion over and above its own signal, and vice-versa. This supports the vector coding results, showing that the encapsulation bra provides the strongest coupling between the breast and torso, i.e. the dynamics of both torso and breast is determined by an external mechanical driver. In comparison, in the compression bra condition, there was variability in breast motion that can be better predicted with knowledge of torso motion, and vice versa. Improvement of predictability of both the breast and the torso motion via knowledge of the other is evident to the highest degree in the no bra condition. Thus, an area of future work is to consider Granger causality with comfort rating of sports bras. Considering aspects of unpredictable movement of the torso and breast during running, alluding to variability in motion and how this variability is coupled between the torso and the breast (which takes into account overall stride variability), it is suggested that levels of comfort might be reflective of variance in stress and strain on tissue over the gait cycle ameliorating the effect of repetitive stress. In this case, finding optimum values relating to comfort is key, where Granger causality could provide a metric to determine this relative motion from empirical data. Furthermore, from a running performance perspective, evidence from musculoskeletal modelling and experimental approaches suggests that increased movement efficiency and running economy occurs in conditions of decreased relative breast to torso motion, due to reductions in extensor spine moments^27,28^.

Complexity in the context of dynamical systems refers to the interdependency of breast and torso dynamics on their subsystems. For example, in the case of the breast the complexity is related to the interaction of the different structural components, including glandular tissue, adipose tissue, connective tissue, blood vessels, and lymphatic vessels. These components work together to provide support, shape, and function to the breast and their interaction determines its collective dynamics. In case of the torso, the components are the musculoskeletal, respiratory, spinal and cardiovascular subsystems. A transfer of complexity between breast and torso would then imply that some of the dynamical characteristics of one would affect the inner dynamics of the other. For example, oscillations or damping due to inertia of the breast could induce a mechanical rhythmical input on the musculoskeletal structure of the torso. Possible secondary effects could include impact on the respiratory system, through changes in the diaphragm rhythmic alternating movement. We examined this transfer of information, or complexity via the measure of transfer entropy.

In line with mechanical understanding, the transfer of information between the breast and torso motion signals was higher when considering the direction of the torso to the breast as opposed to the breast to the torso. However, since no main effect of breast support conditions was observed in the direction of torso to breast, transfer of structural information was relatively unchanged in no bra and bra conditions. This means that the knowledge of the previous movement data of the breast would not improve understanding or predicting future movements of the torso. Presumably this is due to the relatively periodic and constrained motion of the torso in the running action that has higher inertia than that of the breast. Interestingly, transfer of information in the direction of the breast to the torso was significantly higher in the bra conditions compared to the no bra condition, providing evidence that some of the structural characteristics of the breast motion signal are influencing that of the torso when the mechanical constraint of the bra is introduced. Transfer of information from the breast to the torso is indicative of a measurable, mutual influence. Future work might look to establish the relevance of this influence in relation to breast pain and sports performance related factors, through improved modelling of forces acting to and from the breast tissue. This finding of mutual influence of the breast and torso could be incorporated into future modelling studies of torso-breast mechanics and may also be an important factor in investigating the pain experienced in different bra conditions. Furthermore, it may be that nonlinear techniques could be used to examine relative movements in activities where the motion is more random than that of running, for instance while playing sports, since the measures are interested in mutual influence regardless of the motion. Future work is required in all these areas. For example, nonlinear analyses such as transfer entropy may be well placed to quantify the erratic behaviour between the breast and torso previously described in water based running^25^, by providing evidence of the decoupling between torso motion and the breast motion based on nonlinear behaviours. Considering both linear measures of coupling and predictability of the torso-breast system as well as the transfer of complexity of signal structure may help understand differences in breast pain and performance during running with different styles of sports bras.

## Conclusion

In conclusion, breast-torso movement coordination is affected by breast support garments during running with the breast and torso in-phase for ~ 66% of the gait cycle without a bra, compared to over 90% of the gait cycle in either a compression or encapsulation bra. Furthermore, the encapsulation bra produced more in-phase coordination between the breast and torso compared to the compression bra, while more torso dominancy was observed in the compression bra compared to the encapsulation bra. The finding that the breast and torso were almost entirely in-phase across the gait cycle with the encapsulation bra, provides evidence of a ‘locking’ mechanism to move the breast in line with the torso. Since the mid-flight phase and take off phase distinguished the two bra conditions it is suggested that future investigations consider relative phase of breast and torso motion throughout the gait cycle to better define the effects of different forms of breast support and better explain changes in predefined sports bra performance or comfort ratings.

Further supporting the ‘locking’ mechanism of the encapsulation bra, based on Granger causality there is little new information from the torso signal to predict the breast over and above its own motion, and vice-versa. In comparison, in the compression bra condition, there was variability in breast motion that can be better predicted with knowledge of torso motion, and vice versa. We also found evidence of complexity transferred from the breast to the torso in both bra conditions.

Overall, these results provide novel insight into the mutual and complex interaction between the breast and the torso while running in different bra conditions. Our approaches allow for a greater understanding of bra support conditions than existing discrete methods, which may relate to comfort and performance. Therefore, measures of vector coding and Granger causality should be employed in future work examining these features.

Specifically, future work might consider relating levels of breast comfort and support experienced in different bra conditions with the measures of coupling, transfer of information and complexity. In addition, since breast motion is seen to influence torso motion, future work might look to establish the relevance of this influence in relation to breast pain and sports performance related factors, through improved modelling of forces acting to and from the breast tissue.

## Methods

### Participants/data

Prior to the commencement of the study, ethical approval was gained (University of Portsmouth SFEC 2019-031). All participants provided written informed consent before taking part in the study, and all research was performed in accordance with the Declaration of Helsinki.

Twelve healthy female participants (age: 23 years (5 years); mass: 70 kg (6 kg); height: 1.70 m (0.04 m); under bust measurement of 76 cm (1 cm); over bust measurement of 94 cm (1 cm) (mean (SD)) were recruited. Participants were eligible to participate if they were 18 to 39 years of age, a UK bra size 34 D, had not experienced any surgical procedures to the breasts, were not currently undergoing any clinical breast treatments, were not currently pregnant and had not been pregnant or breast-feeding within the last year and were physically active (exercising for more than 30 min at least twice a week). Participants bra size was established using the best-fit bra fitting method^29,30^.

### Data collection

**Fig. 4:**
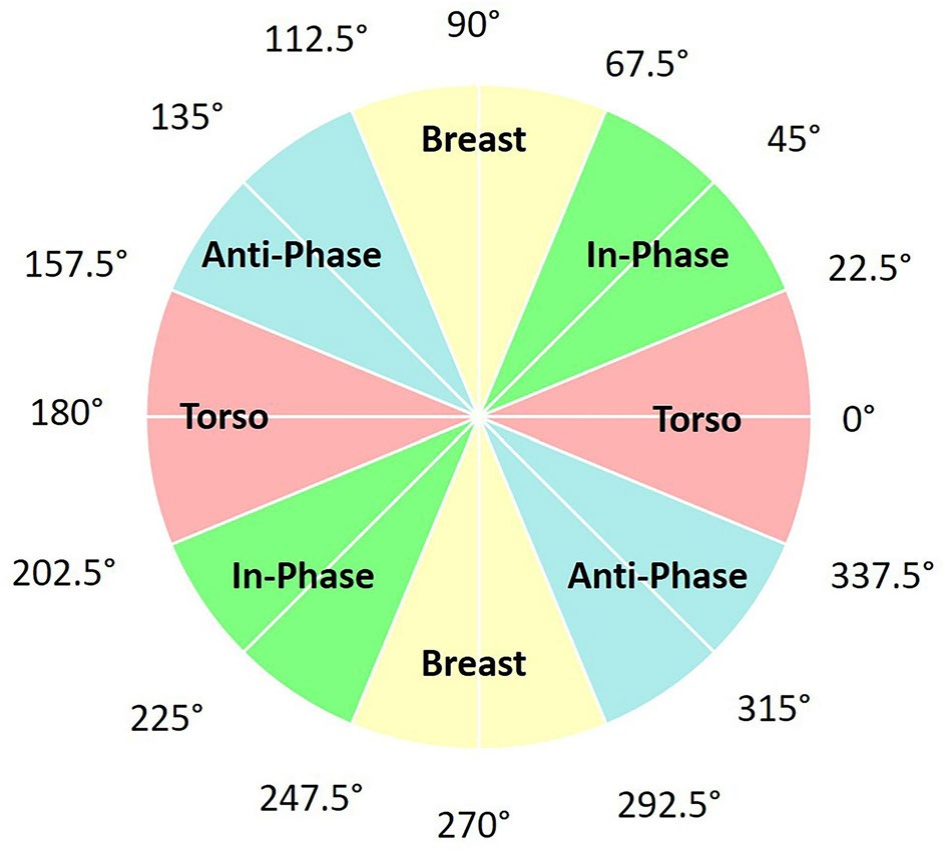
Inter-segment coordination patterns: in-phase, anti-phase, torso dominant phase and breast dominant phase (adapted from Lim et al.^41^).

To measure breast kinematics, nipple and trunk positional data were recorded at 240 Hz using electromagnetic sensors (Micro Sensor 1.8, Liberty, Polhemus, USA) on the suprasternal notch (STN) and left nipple (LNIP). Data from the STN and LNIP were used in the present analysis as a nipple marker has been shown to give a reliable and valid measure of breast displacement^31^ and the left breast frequently moves more during running than the right breast^32^ (Fig. 4).

**Fig. 5:**
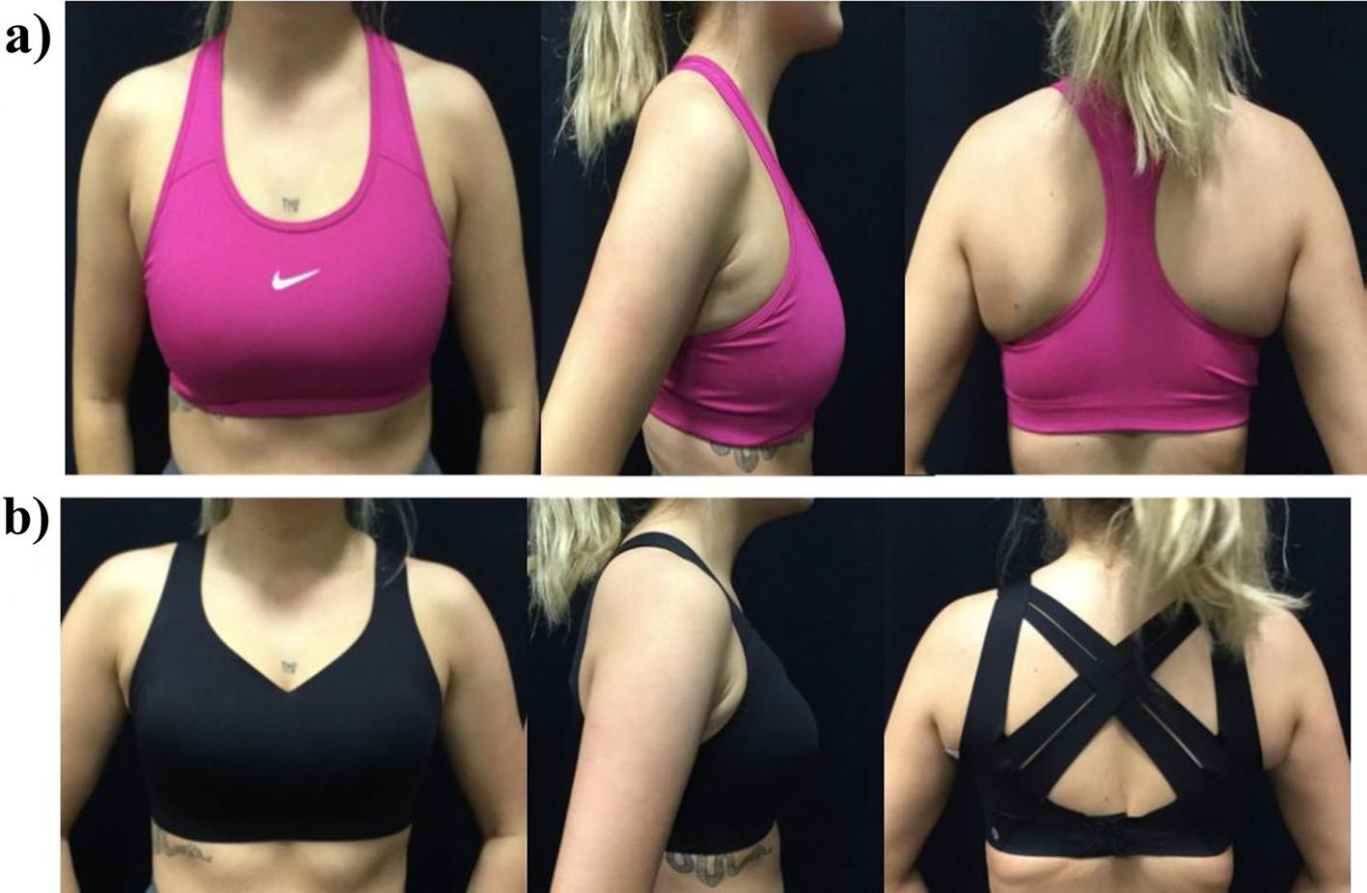
(**a**) Compression style sports bra (Nike, Dri-FIT Swoosh Women's Medium-Support 1-Piece Pad Sports Bra) (**b**) High support encapsulation style sports bra (Lululemon, Enlite).

Participants performed a one-minute warm-up on a treadmill (H/P/Cosmos Mercury, Germany) at a self-selected speed. Participants then ran at 10 km/h for up to a minute in two sports bras and with no bra and data were recorded for 30 s in the sports bras and 20 s with no bra^5^. A bare-breasted (no bra) static trial was recorded in the anatomical position for 10 s^33,34^. In order to test sports bras with an expected difference in level of support a compression style sports bra (Nike, Dri-FIT Swoosh Women's Medium-Support 1-Piece Pad Sports Bra) and an encapsulation style sports bra (Lululemon, Enlite High Support Sports Bra) were selected (Fig. 5). The encapsulation bra is a high-end sports bra marketed for “high-impact activities” and “designed for running”, whereas the compression bra is more modestly priced and marketed as providing “medium-impact support for activities like spin, cardio and dance classes”. The order of the bra conditions were randomised, however the no bra condition was always performed last due to the discomfort in the no bra condition which may have carried over a bra condition.

The data that support the findings of this study are available in [repository name] at [URL/DOI], reference number [reference number].

### Data Processing

Data were converted into .c3d files and processed in Visual3D (Version 6, C-Motion, Inc., Germantown, MD, USA), from which they were exported to text files and further processed in MATLAB (Version R2017b, MathWorks Inc., USA). Data were analysed in the vertical direction in the global coordinate system, except for the traditional kinematics. Data were filtered using a Lowpass Butterworth filter at 13 Hz^32,35,36^.

### Dividing the signals into gait cycles

Recent breast biomechanics work has not distinguished between the left and right foot strike^1,32–34^.This could lead to an under or over estimation of biomechanical variables and it is vital to be able to distinguish between the left and the right foot in order to investigate nipple kinematics across the entire the gait cycle. For the calculation of the coupling methods, it was necessary to estimate instances of the gait cycle/identify contralateral or ipsilateral foot relative to the breast being measured. To do this, signals were divided into cycles starting from left foot strike based on STN position. This resulted in an average of 42 cycles in the bra conditions and an average of 27 cycles in the no bra condition. Maximum and minimum medio-lateral position of STN was used to discriminate between left and right foot contact^37^. Based on an analysis of previous breast optical motion capture kinematics in bare-breasted no-bra running, it was estimated that the minimum vertical position of STN occurred ~ 100 ms after foot contact^38^. Therefore, left foot contact was defined as the minimum vertical position of STN which occurred adjacent to peak medio-lateral position of STN, minus 100 ms.

To place the vector coding results in context of traditional biomechanical measurements, we calculated discrete time lag.

### Discrete time-lag

Discrete time-lag was calculated as the average difference per gait cycle between the most superior nipple position and the most superior torso position as a percentage of the gait cycle^4,39^.

### Vector coding

**Fig. 6:**
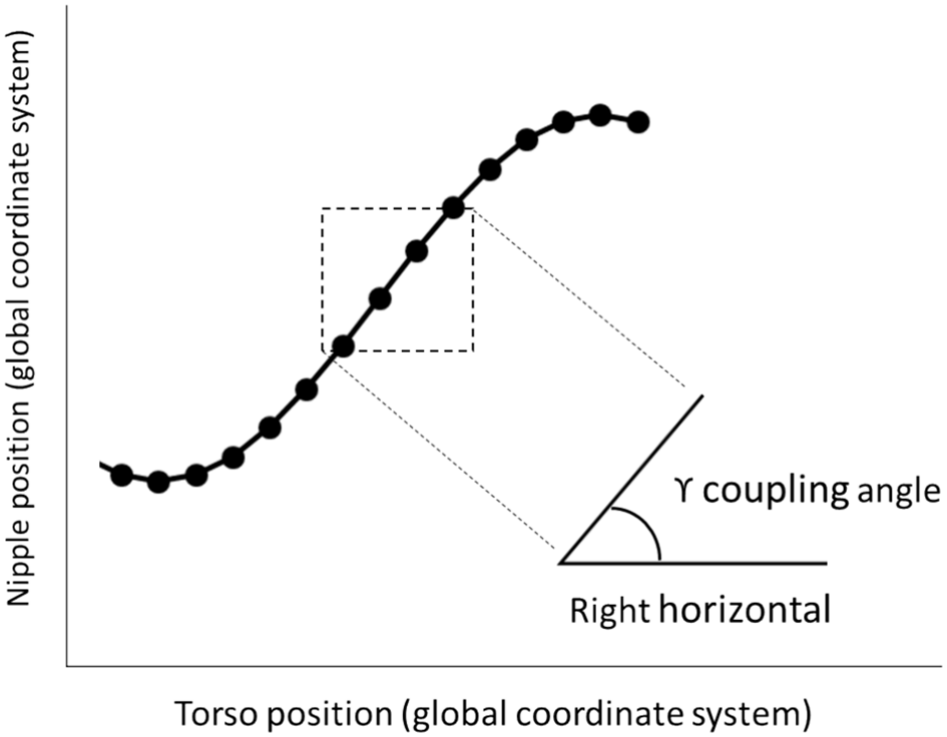
Definition of vector coding coupling angle relative to the right horizontal (adapted from Lim et al.^41^).

Vector coding (phase angle) was calculated based on the first and second oscillator movements V1 and V2, according to the following Eq. (1):

1 $$\theta (i) = {\tan^{ - 1}}\left( {\frac{{{V_2}(i + 1) - {V_2}(i)}}{{{V_1}(i + 1) - {V_1}(i)}}} \right)$$

where 0° ≤ θ ≤ 360° and i represents consecutive data points. Figure 4 represents the interpretation of phase angles based on the definition presented in Fig. 6 and Eq. (1). Mean coupling angles were calculated using circular statistics^40^. Coordination patterns were categorised into bins of breast dominancy, torso dominancy, in-phase and anti-phase (Fig. 4)^8^.

### Granger causality

Granger causality^19^ was calculated by applying its definition:

$$GC(X \to Y) = \ln (Sigma\,1/Sigma\,2)$$

where Sigma1 is the variance of the linear regression considering just past values of Y alone, and Sigma2 is the variance of the linear regression considering past values of both X and Y (in our case, torso and nipple displacement). The computation was made using a custom-made function in RStudio on the entire filtered signals for 10 gait cycles in each bra condition. If there were no gain in including past values of both X and Y in the prediction of future value of Y, Sigma1 = Sigma2, and GC(X- > Y) = ln(1) = 0. The higher the value, the greater the benefit.

### Transfer entropy

To determine information transfer between two time series, in this case the torso and the nipple motion, transfer entropy considers the conditional mutual information and it is model-free, allowing the measurement of time-directed transfer of information between stochastic variables without assuming linearity^42^.Transfer entropy was calculated based on RTransferEntropy functions in RStudio (RStudio, PBC, Boston, MA). The number of bootstrap replications for each direction of the estimated transfer entropy was set to 300, the number of shuffles used to calculate the effective transfer entropy was 100, and the quantiles of the empirical distribution of the respective time series used for discretisation were 5, 95. For example, a transfer entropy of 0.01 means that the history of the X (for example the torso) process has 0.01 bits of additional information for the prediction of the next value of Y (for example the nipple). (i.e., it provides information about the future of Y, in addition to what we know from the history of Y). The higher the value of transfer entropy, the higher the information that is transferred from the time history of X to Y and visa-versa.

### Statistical analysis

Discrete statistical analysis was performed in SPSS (Version 28, IBM, Chicago, IL). The distribution of data was inspected visually from the skew in the histograms. Differences in discrete variables between breast support conditions were compared using one-way repeated measures ANOVA (α = 0.05). Coordination pattern frequencies over the gait cycle were compared for each of the four frequency bins. Data were tested for sphericity using Mauchly’s test. A Bonferroni post hoc analysis was applied to investigate significant main effects. Statistical parametric mapping (SPM) was used to compare mean vector coding angle waveforms of each bra support condition across the gait cycle^18,43^.

## Data Availability

The datasets generated during and/or analysed during the current study are available in the University of Exeter Repository (ORE). For any other data query, please get in touch with the corresponding author.
